# Depression mediates the association of healthy sleep patterns with suicidal ideation among U.S. adults

**DOI:** 10.3389/fpsyt.2025.1644867

**Published:** 2025-09-29

**Authors:** Xue Yang, Chao Ning, Rong Liu, Shanshan Zhang, Yi Gong, Qingping Xue, Jieru Peng, Shiyi Wu, Yanan Wang

**Affiliations:** ^1^ Department of Endocrinology, The First Affiliated Hospital of Xi’an Jiaotong University, Xi’an, China; ^2^ Med-X Institute, Center for Immunological and Metabolic Diseases, the First Affiliated Hospital of Xi’an Jiaotong University, Xi’an, China; ^3^ Shaanxi Provincial Centre for Disease Control and Prevention, Xi’an, China; ^4^ Department of Epidemiology and Biostatistics, West China School of Public Health and West China Fourth Hospital, Sichuan University, Chengdu, China; ^5^ Department of Epidemiology and Biostatistics, School of Public Health, Chengdu Medical College, Chengdu, Sichuan, China

**Keywords:** sleep patterns, depression, suicidal ideation, mediation, suicide prevention

## Abstract

**Background:**

Sleep disturbances are common and strongly linked to depression and suicidal ideation, both of which are major public health concerns. However, evidence on whether depression mediates the association between overall sleep patterns and suicidal ideation remains limited.

**Methods:**

We conducted a cross-sectional analysis using data from 5,978 U.S. adults participating in the National Health and Nutrition Examination Survey. Participants were included if they completed the sleep, depression, and suicidal ideation assessments. A composite sleep score was constructed from four distinct sleep behaviors. Depression was defined as the Patient Health Questionnaire-9 (PHQ-9) score of ≥10, and suicidal ideation was assessed by the ninth PHQ-9 item (score 1-3). Logistic regression was used to evaluate associations between sleep score and depression/suicidal ideation, and mediation analysis tested the indirect effect of depression.

**Results:**

The mean age of participants was 45.00 years, and 52.85% were men. Higher sleep scores were linearly associated with lower odds of both depression and suicidal ideation (*P* for trend < 0.001). Compared with participants scoring 0-1, those scoring 4 had markedly reduced odds of depression (odds ratio [OR], 0.12; 95% confidence interval [CI], 0.06, 0.25) and suicidal ideation (OR, 0.20; 95% CI, 0.12, 0.36). Depression partially mediated the association between the healthy sleep score and suicidal ideation, accounting for 36.2% of the effect.

**Conclusions:**

Healthy sleep patterns are strongly associated with lower likelihoods of depression and suicidal ideation. These findings highlight the clinical importance of promoting healthy sleep behaviors and integrating depression screening and management into suicide prevention strategies.

## Introduction

Suicide is a major global public health challenge, accounting for more than 700,000 deaths annually (1). In the United States alone, 49,449 people died from suicide in 2022, representing a 2.6% increase from 2021 (2). Suicidal ideation, which reflects the initial stage of suicidal behavior, is a strong predictor of subsequent suicide attempts and deaths, even though not all individuals with suicidal ideation progress to these outcomes (3). Identifying modifiable risk factors for suicidal ideation is therefore essential for effective suicide prevention.

Sleep plays a fundamental role in physical and mental health. Disturbances in sleep have been associated not only with chronic conditions such as type 2 diabetes and cardiovascular disease (4, 5), but also with increased risks of depression and suicidal behaviors, including suicidal ideation, suicide attempts, and completed suicide (6). These findings underscore the importance of sleep as a potential upstream factor in the pathway leading to suicidal risk. Instead of focusing on single sleep dimensions such as insomnia or sleep duration, recent research has emphasized the value of composite sleep patterns that incorporate multiple interrelated behaviors (e.g., duration, insomnia, snoring, daytime sleepiness) (7–10). This multidimensional approach better reflects the complexity of sleep health and may provide a more accurate understanding of its impact on mental well-being.

Depression, one of the most prevalent psychiatric disorders worldwide, affects nearly 5% of the global population (11). In the United States, 18.4% of adults had been diagnosed with depression during 2020 (12). Characterized by persistent sadness, hopelessness, and diminished interest, depression impairs daily functioning and quality of life, and is among the strongest predictors of suicidal ideation and behavior (3). Importantly, depression is closely linked to sleep: poor sleep is a well-established risk factor for depression, and depression in turn substantially increases the likelihood of suicidal ideation (7, 13–15). This interconnection provides a strong theoretical basis for considering depression as a potential mediator in the relationship between sleep and suicidal ideation.

Despite these established associations, most prior studies have examined individual sleep behaviors in isolation, limiting insights into the joint impact of overall sleep patterns on depression and suicidal ideation. Moreover, few studies have empirically tested whether depressive symptoms explain, at least partly, the link between unhealthy sleep patterns and suicidal ideation in large population-based settings. Addressing this gap is critical for understanding the underlying mechanisms and informing effective prevention strategies.

Therefore, the present study aimed to (1) examine the association between a comprehensive healthy sleep score and suicidal ideation in the general population, and (2) assess whether depressive symptoms mediate this association. By integrating sleep, depression, and suicidal risk into a unified framework, our study provides novel evidence for the pathways linking sleep health to suicide prevention.

## Materials and methods

### Design of the study and participants

The National Health and Nutrition Examination Survey (NHANES) is an ongoing study focusing on the general population. The survey employed a multi-stage, stratified probability sampling design to ensure a representative sample of the U.S. population. The NHANES was commenced before 1999 and has constantly collected diverse information, encompassing data from in-home interviews, mobile examinations, and laboratory tests. All assessments in NHANES were conducted by trained interviewers with professional backgrounds in health-related fields. Interviewers received standardized training in questionnaire administration and the handling of sensitive topics, and their performance was monitored by field supervisors to ensure data quality and consistency. The NHANES was conducted in accordance with the Declaration of Helsinki and approved by the National Center for Health Statistics Research Ethics Review Board. All participants provided written informed consent and received a small token of appreciation as part of the standard NHANES protocol.

Survey data from NHANES 2005–2006 and 2007–2008 were used in our study due to their inclusion of the necessary information for assessing the healthy sleep score. Initially, 20,497 U.S. adults were eligible for our study. Nevertheless, we excluded specific participants from our study, including individuals younger than 18 years (n=8,706), individuals without comprehensive data on sleep evaluation (n=1,337), and individuals who were taking sleeping tablets (n=1,994). We also excluded individuals with missing data on suicidal ideation (n=1,113) or depression (n=43), and individuals without sociodemographic information such as education (n=639), marital status (n=4), poverty-to-income ratio (PIR) (n=434), body mass index (BMI) (n=57), cigarette smoke (n=3), alcohol consumption (n=16), physical activity (n=68), hypertension (n=82), diabetes (n=36), and dyslipidemia (n=207). Finally, a total of 5,978 participants were included in the analyses (**Supplementary Figure 1**).

### Evaluation of sleep behaviors and definition of healthy sleep behaviors

Sleep behaviors, including sleep duration, insomnia, snoring, and daytime sleepiness, were assessed using single self-reported questions through the in-home interviews. Previous validation studies comparing this NHANES single-item measure with wrist-worn accelerometer data indicate that self-reported sleep tends to slightly overestimate actual sleep duration, but it remains useful for large-scale population studies (16, 17). In our study, sleep duration was determined by the question, “How much sleep do you usually get at night on weekdays or workdays?” Insomnia was evaluated through three questions: 1) “In the past month, how often did you have trouble falling asleep?”; 2) “In the past month, how often did you wake up during the night and have trouble getting back to sleep?”; 3) “In the past month, how often did you wake up too early in the morning and were unable to get back to sleep?”, with response options: “Never,” “Rarely (1 time a month),” “Sometimes (2–4 times a month),” “Often (5–15 times a month),” and “Almost always (16–30 times a month).” Snoring was assessed with the question, “In the past 12 months, how often did you snore while sleeping?” using the response categories: “Never,” “Rarely (1–2 nights/week),” “Occasionally (3–4 nights/week),” and “Frequently (5 or more nights/week).” Lastly, information regarding excessive daytime sleepiness was collected using the question, “In the past month, how often did you feel excessively or overly sleepy during the day?” with the same five response categories as insomnia.

Low-risk sleep behaviors were defined as follows: sleep duration of 7–8 hours per day, no frequent insomnia (never, rarely, and occasionally), no snoring (never and rarely), and no excessive daytime sleepiness (never, rarely, and sometimes). For each sleep behavior, the participants got a score of 1 if she/he met the low-risk criteria or 0 if not. Then, all low-risk scores were summed to obtain the comprehensive healthy sleep score, which ranged from 0 to 4. A higher score indicated a more favorable sleep pattern (18).

### Assessment of depression and suicidal ideation

The Patient Health Questionnaire-9 (PHQ-9), a widely utilized instrument for assessing mental health over the previous two weeks, was used to evaluate depression in our study (19). Nine criteria from the Diagnostic and Statistical Manual of Mental Disorders are included in this scale, which has a score range of 0 (not at all) to 3 (almost every day). The overall score, ranging from 0 to 27, reflects the cumulative points of these nine categories and represents the severity of depression (19). The PHQ-9 is a well-validated tool, and a score of ≥ 10 indicates an 88% sensitivity and 85% specificity in detecting major depression during screening (20). Hence, participants in this study were considered to have depression if their PHQ-9 scores were 10 or higher. Internal consistency of the PHQ-9 was also evaluated in our sample using Cronbach’s *α* to assess reliability.

The assessment of suicidal ideation entailed participants’ answers to item #9 of the PHQ-9 questionnaire: “Over the last 2 weeks, how often have you been bothered by the following problem: Thoughts that you would be better off dead or of hurting yourself in some way?”, which inquired about the frequency of suicidal ideation throughout the previous fortnight (not at all=0, several days=1, more than half=2, and nearly every day=3). Suicidal ideation was characterized by a score ranging from 1 to 3 (21). The focus of our study was limited to suicidal ideation rather than suicidal attempt. Within the NHANES dataset, item #9 of the PHQ-9 represented the only available measure of suicidal ideation. Although other instruments specifically designed to assess suicidal ideation and attempts exist, the PHQ-9 has been extensively validated and widely applied in both clinical and epidemiological research, including large-scale population-based cohorts (22). Its use in the present study ensured methodological consistency with prior work and comparability across studies employing NHANES data.

### Measurement of covariates

On the basis of earlier studies, potential confounders were identified. Sociodemographic factors, lifestyle and behaviors, and medical problems data were collected via in-home interviews. Anthropometric measurements were conducted in the mobile examination center to evaluate the height, weight, and blood pressure. The BMI was calculated by dividing the weight, measured in kilograms, by the square of the height, measured in meters. The participants were classified into three groups based on their smoking behaviors. Individuals who had consumed less than 100 cigarettes before the study were classified as never-smokers. Individuals who had previously ceased smoking before the study but had consumed over 100 cigarettes were classified as ever smokers. Individuals who declared that they were currently smoking during the survey were classified as current smokers. Alcohol consumption was classified into three categories: moderate drinkers (<12 drinks/year), heavy drinkers (≥1 drink/day for women and ≥2 drinks/day for men), and non-drinkers (<12 drinks/year). Physical activity levels were classified into three categories based on the weekly duration of moderate-to-intense exercise: light activity (less than 150 minutes per week), moderate activity (150 to 300 minutes per week), and heavy activity (more than 300 minutes per week). Hypertension was deemed to be present if participants were using antihypertensive drugs or if their blood pressure readings were ≥140/90 mm Hg (23). Diabetes was diagnosed with the self-reported usage of antidiabetic medications or if the fasting blood glucose was ≥126 mg/dL or if the glycated hemoglobin was equal to or higher than 6.5% (24). Participants who were on lipid-lowering medications or had non-high-density lipoprotein cholesterol levels equal to or higher than 160 mg/dL were classified with dyslipidemia (25).

### Patient and public involvement statement

It was not appropriate or possible to involve patients or the public in the design, or conduct, or reporting, or dissemination plans of our research.

### Statistical analysis

The Rao-Scott chi-square test and student’s *t*-test were separately performed to compare categorical and continuous characteristics between two groups, accounting for sampling weights. Basic characteristics were reported across the diagnosis of depression and suicidal ideation, respectively. Furthermore, we compared the basic characteristics across various levels of the healthy sleep score.

We performed multivariable-adjusted logistic regression to calculate the odds ratios (ORs) and 95% confidence intervals (CIs) associated with the healthy sleep score. Three regression models were fitted. Model 1 considered the NHANES cycle, age, sex, race, education, marital status, and PIR as covariates. In Model 2, we incorporated additional covariates, including BMI, cigarette smoke, alcohol consumption, and physical activity. Model 3 was further adjusted for long-term conditions such as dyslipidemia, diabetes, and hypertension. The *P* values for the trend were calculated by modeling the healthy sleep score as an ordinal variable in the regression models. When examining the associations of individual sleep behaviors with depression and suicidal ideation, the covariates were the same as those controlled in the primary analyses.

In addition, we conducted additional stratification of the main analyses to assess the associations of the healthy sleep score with depression and suicidal ideation based on various factors such as age group, sex, obesity status, physical activity, hypertension, diabetes, and dyslipidemia. Interaction terms were added to the fully adjusted model to test any interactions between the healthy sleep score and the stratified factors, and the Wald test was used to test their significance. We then conducted mediation analyses to assess the role of depression in the associations of healthy sleep score and individual sleep behaviors with suicidal ideation. To ensure the validity of the mediation analysis, we examined the statistical assumptions of both the mediation and outcome models. Multicollinearity was assessed using the variance inflation factor (VIF), with values <5 considered acceptable. The distribution of residuals was evaluated using skewness, kurtosis, and Q-Q plots. Heteroscedasticity was assessed by the Breusch-Pagan test. All diagnostic analyses were performed for both the mediation (depression regressed on sleep pattern and covariates) and the outcome model (suicidal ideation regressed on sleep pattern, depression, and covariates). Since the residuals did not fully meet the normality assumption, we additionally estimated the mediation effects using a quasi-Bayesian approximation with 5,000 simulations to provide robust inference.

Several sensitivity analyses were conducted to guarantee the reliability of the relationships observed in the main analyses. First, the correlation between the healthy sleep score and the depressive score was examined. Second, to further examine the impact of depression on the relationship between the healthy sleep score and suicidal ideation, we further controlled for the depression status in the fully adjusted model. Third, to address potential bias due to missing sociodemographic data and assess the robustness of our primary findings, we conducted a sensitivity analysis using multiple imputations for 7,524 participants. Participants with missing data on sociodemographic variables (e.g., education, marital status, poverty-to-income ratio, BMI, smoking and drinking status, physical activity) were included in this analysis rather than excluded. In addition, given the potential confounding effect of antidepressant treatment, two further sensitivity analyses were performed. We first excluded participants who reported antidepressant medication use and re-estimated the associations between sleep patterns, depression, and suicidal ideation. We then included antidepressant medication use as an additional covariate in the fully adjusted model (Model 3).

We used R 4.2.1 software for all analyses. A two-tailed *P* < 0.05 was considered statistically significant. Throughout our statistical analyses, we employed survey estimation commands such as the “svyglm” function to fully account for the multi-stage and complicated sample design utilized by NHANES.

## Results

### Basic characteristics

The average age of the 5,978 U.S. adults was 45.00 years, with a standard error of 0.44. Additionally, 52.82% of the participants were men, taking into account the sample weights. Depression was found to affect 5.84% of the population, while suicidal ideation affected 2.71%. Adults with suicidal ideation exhibited a greater likelihood of being Hispanic, separated, current smokers, and having lower education levels, lower PIR, lighter physical activity, lower healthy sleep score, and higher prevalence of diabetes compared to those without suicidal ideation (all *P* values ≤0.039; **Table 1**). Except for age and BMI, the distribution of the aforementioned characteristics among individuals with and without depression was comparable to that of suicidal ideation. Nevertheless, people diagnosed with depression had a greater likelihood of being female and having a higher BMI (both *P* values < 0.001; **Table 1**). The distribution of basic characteristics across the healthy sleep score groups was shown in **Supplementary Table 1**. Individuals who had higher healthy sleep scores were more likely to be female, Hispanic, highly educated, unmarried, non-smokers, and have higher PIR, lower BMI, and a reduced prevalence of hypertension, diabetes, and dyslipidemia (all *P* values ≤0.006;**Supplementary Table 1**). The PHQ-9 scores in the current sample had a mean of 2.55 (SD = 3.60). The scale demonstrated good internal consistency, with Crobach’s *α* = 0.83 (data not shown).

**Table 1:** Characteristics of study participants in NHANES, 2005-2008 (N = 5,978).

Characteristics	Overall	Depression			Suicidal ideation		
		Without	With	*P* value ^a^	Without	With	*P* value ^a^
No. of participants, n (%)	5,978 (100.00)	5,629 (94.16)	349 (5.84)	–	5,816 (97.29)	162 (2.71)	–
Age, years	45.00 ± 0.44	45.01 ± 0.45	43.81 ± 0.94	0.228	44.98 ± 0.44	43.88 ± 1.33	0.406
Sex, n (%)				<0.001			0.802
Male	3,129 (52.82)	2,987 (53.43)	142 (40.36)		3,054 (52.84)	75 (51.58)	
Female	2,849 (47.18)	2,642 (46.96)	207 (59.64)		2,762 (47.16)	87 (48.42)	
Race, n (%)				0.002			<0.001
Hispanic	1,586 (12.69)	1,475 (12.45)	111 (17.52)		1,515 (12.29)	71 (31.09)	
Non-Hispanic white	2,885 (71.10)	2,747 (71.64)	138 (60.21)		2,833 (71.53)	52 (51.39)	
Non-Hispanic black	1,278 (10.66)	1,191 (10.39)	87 (16.18)		1,244 (10.62)	34 (12.71)	
Others	229 (5.55)	216 (5.52)	13 (6.10)		224 (5.57)	5 (4.81)	
Education, n (%)				<0.001			0.001
Below high school	1,671 (18.14)	1,519 (17.42)	152 (32.75)		1,595 (17.80)	76 (33.68)	
High school	1,428 (24.08)	1,336 (23.69)	92 (32.12)		1,392 (24.05)	36 (25.64)	
College and above	2,879 (57.78)	2,774 (58.89)	105 (35.13)		2,829 (58.16)	50 (40.68)	
Marital status, n (%)				<0.001			<0.001
Married	3,461 (60.72)	3,320 (61.62)	141 (42.39)		3,402 (61.27)	59 (35.31)	
Separated	1,595 (23.54)	1,457 (22.75)	138 (39.55)		1,530 (23.14)	65 (41.65)	
Never married	922 (15.74)	852 (15.63)	70 (18.06)		884 (15.59)	38 (23.04)	
PIR, n (%)				<0.001			<0.001
<1.3	1,610 (17.32)	1,432 (16.15)	178 (41.19)		1,525 (16.82)	85 (40.52)	
1.3-3.5	2,387 (37.07)	2,261 (37.05)	126 (37.46)		2,325 (36.87)	62 (46.43)	
>3.5	1,981 (45.61)	1,936 (46.80)	45 (21.35)		1,966 (46.32)	15 (13.04)	
Body mass index, kg/m^2^	28.68 ± 0.18	28.60 ± 0.18	30.40 ± 0.57	0.004	28.66 ± 0.18	29.76 ± 0.86	0.205
Obesity status, n (%)				0.005			0.260
Normal	1,705 (31.10)	1,614 (31.24)	91 (28.18)		1,664 (31.19)	41 (26.81)	
Overweight	2,122 (34.89)	2,021 (35.26)	101 (27.29)		2,066 (34.94)	56 (32.80)	
Obesity	2,151 (34.01)	1,994 (33.50)	157 (44.53)		2,086 (33.88)	65 (40.38)	
Cigarette smoke, n (%)				<0.001			<0.001
Never	3,215 (53.31)	3,067 (53.96)	148 (40.20)		3,138 (53.41)	77 (48.88)	
Ever	1,488 (23.91)	1,419 (24.25)	69 (16.83)		1,465 (24.18)	23 (11.32)	
Current	1,275 (22.78)	1,143 (21.79)	132 (42.97)		1,213 (22.41)	62 (39.80)	
Alcohol consumption, n (%)				0.643			0.236
None	1,760 (24.81)	1,658 (24.69)	102 (27.40)		1,705 (24.70)	55 (29.90)	
Moderate	3,706 (65.32)	3,492 (65.47)	214 (62.34)		3,619 (65.54)	87 (55.44)	
Heavy	512 (9.86)	479 (9.84)	33 (10.26)		492 (9.76)	20 (14.66)	
Physical activity, n (%)				<0.001			0.039
Light	2,307 (32.97)	2,118 (32.13)	189 (50.11)		2,227 (32.71)	80 (44.93)	
Moderate	1,763 (32.88)	1,690 (33.28)	73 (24.79)		1,724 (32.93)	39 (30.48)	
Heavy	1,908 (34.15)	1,821 (34.60)	87 (25.10)		1,865 (34.36)	43 (24.59)	
Hypertension, yes, n (%)	2,533 (37.41)	2,378 (37.25)	155 (40.69)	0.225	2,461 (37.30)	72 (42.71)	0.213
Diabetes, yes, n (%)	877 (10.47)	797 (10.12)	80 (17.69)	0.003	839 (10.25)	38 (20.53)	0.002
Dyslipidemia, yes, n (%)	2,649 (42.38)	2,478 (42.13)	171 (47.33)	0.105	2,585 (42.50)	64 (36.82)	0.360
Healthy sleep score	2.66 ± 0.02	2.70 ± 0.02	1.71 ± 0.09	<0.001	2.67 ± 0.02	1.94 ± 0.12	<0.001

NHANES, National Health and Nutrition Examination Survey; PIR, poverty-to-income ratio.

a *P* values were calculated using Rao–Scott Chi-square test and student’s t-test for categorical and continuous variables.

Data was presented as mean ± standard error (continuous variables) and number (percent) (categorical variables).

### Associations of the healthy sleep score with depression and suicidal ideation

Multivariable-adjusted logistic regression models showed negative associations between the healthy sleep score and depression (all *P* for trend <0.001; **Table 2**). The ORs (95% CIs) for depression in persons with a sleep score of 4 in the three models were 0.10 (0.05, 0.18), 0.11 (0.06, 0.22), and 0.12 (0.06, 0.25), respectively, compared to the reference group with a sleep score of 0-1 (**Table 2**). Model 3 showed that with each additional improvement in the healthy sleep score, the likelihood of experiencing depression decreased by 52% (OR: 0.48; 95% CI: 0.41, 0.56; **Table 2**).

**Table 2:** The association of healthy sleep score with depression and suicidal ideation in NHANES, 2005-2008.

	Healthy sleep score: OR (95% CI)				*P* for trend	Per 1 point increment
	0-1	2	3	4		
Depression						
Cases/participants	146/850	111/1,568	68/2,251	24/1,309	–	–
Model 1	1.00	0.43 (0.31, 0.59)	0.14 (0.09, 0.22)	0.10 (0.05, 0.18)	<0.001	0.46 (0.40, 0.53)
Model 2	1.00	0.45 (0.32, 0.63)	0.15 (0.09, 0.24)	0.11 (0.06, 0.22)	<0.001	0.47 (0.41, 0.55)
Model 3	1.00	0.46 (0.32, 0.66)	0.15 (0.09, 0.25)	0.12 (0.06, 0.25)	<0.001	0.48 (0.41, 0.56)
Suicidal ideation						
Cases/participants	56/850	48/1,568	42/2,251	16/1,309	–	–
Model 1	1.00	0.43 (0.24, 0.77)	0.26 (0.15, 0.44)	0.18 (0.10, 0.31)	<0.001	0.56 (0.47, 0.68)
Model 2	1.00	0.43 (0.24, 0.79)	0.27 (0.16, 0.46)	0.20 (0.12, 0.33)	<0.001	0.57 (0.48, 0.68)
Model 3	1.00	0.45 (0.23, 0.88)	0.28 (0.15, 0.50)	0.20 (0.12, 0.36)	<0.001	0.58 (0.48, 0.70)

CI, confidence interval; NHANES, National Health and Nutrition Examination Survey; OR, odds ratio.

Model 1: adjusted for NHANES cycles, age (continuous, year), sex (male and female), race (Hispanic, non-Hispanic white, non-Hispanic black, and others), education (below high school, high school, and college and above), marital status (married, separated, and never married), and poverty-to-income ratio (<1.3, 1.3-3.5, and >3.5);

Model 2: Adjusted for body mass index (continuous, kg/m^2^), cigarette smoke (never, ever, and current), alcohol consumption (none, moderate, and heavy), physical activity (light, moderate, and heavy), and covariates adjusted in Model 1;

Model 3: Adjusted for hypertension (yes and no), diabetes (yes and no), dyslipidemia (yes and no), and covariates adjusted in Model 2.

Similarly, a healthy sleep score was inversely associated with the likelihood of suicide ideation. In Model 1, we found a negative correlation between the healthy sleep score and suicidal ideation, with a dose-response pattern (*P* for trend <0.001; **Table 2**). The inverse association was marginally weakened in Model 2 after adjusted for lifestyle and behaviors and further weakened in Model 3 with adjustment for chronic conditions. When compared to the reference group, with a healthy sleep score of 0-1, people with a healthy sleep score of 4 had ORs (95% CIs) for suicidal ideation of 0.18 (0.10, 0.31), 0.20 (0.12, 0.33), and 0.20 (0.12, 0.36) in the three models, respectively (**Table 2**). In the fully adjusted model, there was a significant association between a 1-point increase in the healthy sleep score and a 42% decreased likelihood of suicidal ideation (0.58; 0.48, 0.70; **Table 2**).

Furthermore, we performed subgroup analyses to examine the potential influence of stratification factors on the associations between the healthy sleep score and depression, as well as suicidal ideation. No significant interaction was observed between the healthy sleep score and the stratified factors (i.e. age, sex, physical activity, and disease conditions) in relation to depression and suicidal ideation (all *P* values for interaction ≥0.055; **Table 3**).

**Table 3:** Subgroup analysis of the association of healthy sleep score with depression and suicidal ideation.

Subgroup	Depression					Suicidal ideation				
	Healthy sleep score: OR (95% CI)				*P* for interaction ^a^	Healthy sleep score: OR (95% CI)				*P* for interaction ^a^
	0-1	2	3	4		0-1	2	3	4	
Age					0.120					0.699
≤ 60 years	1.00	0.49 (0.33, 0.72)	0.15 (0.08, 0.29)	0.14 (0.06, 0.31)		1.00	0.46 (0.23, 0.94)	0.27 (0.13, 0.54)	0.21 (0.11, 0.42)	
> 60 years	1.00	0.43 (0.19, 0.95)	0.17 (0.07, 0.45)	0.02 (0.00, 0.12)		1.00	0.52 (0.10, 2.67)	0.53 (0.13, 2.14)	0.23 (0.03, 2.07)	
Sex					0.628					0.126
Male	1.00	0.37 (0.20, 0.66)	0.13 (0.07, 0.25)	0.12 (0.04, 0.31)		1.00	0.73 (0.34, 1.59)	0.40 (0.18, 0.90)	0.29 (0.13, 0.68)	
Female	1.00	0.54 (0.32, 0.91)	0.16 (0.08, 0.34)	0.11 (0.04, 0.28)		1.00	0.26 (0.10, 0.69)	0.19 (0.07, 0.50)	0.14 (0.05, 0.39)	
Obesity status					0.229					0.055
Normal	1.00	0.38 (0.17, 0.85)	0.07 (0.03, 0.16)	0.12 (0.05, 0.30)		1.00	0.83 (0.28, 2.43)	0.30 (0.09, 0.98)	0.49 (0.16, 1.48)	
Overweight	1.00	0.51 (0.27, 0.96)	0.12 (0.05, 0.31)	0.12 (0.04, 0.35)		1.00	0.52 (0.24, 1.13)	0.17 (0.05, 0.57)	0.18 (0.06, 0.53)	
Obesity	1.00	0.43 (0.26, 0.72)	0.23 (0.13, 0.39)	0.07 (0.02, 0.21)		1.00	0.30 (0.09, 0.99)	0.41 (0.16, 1.05)	0.03 (0.00, 0.18)	
Physical activity					0.277					0.396
Low	1.00	0.38 (0.16, 0.94)	0.27 (0.11, 0.66)	0.14 (0.03, 0.60)		1.00	0.56 (0.35, 0.90)	0.21 (0.10, 0.42)	0.10 (0.04, 0.31)	
Moderate	1.00	0.39 (0.12, 1.35)	0.09 (0.01, 0.55)	0.21 (0.04, 1.13)		1.00	0.30 (0.13, 0.66)	0.09 (0.03, 0.26)	0.14 (0.04, 0.53)	
Heavy	1.00	0.55 (0.18, 1.73)	0.54 (0.20, 1.44)	0.15 (0.04, 0.64)		1.00	0.50 (0.26, 0.95)	0.12 (0.05, 0.29)	0.09 (0.03, 0.28)	
Hypertension					0.070					0.677
No	1.00	0.48 (0.27, 0.85)	0.14 (0.08, 0.26)	0.15 (0.07, 0.33)		1.00	0.51 (0.19, 1.36)	0.29 (0.11, 0.79)	0.26 (0.10, 0.69)	
With	1.00	0.45 (0.25, 0.80)	0.16 (0.09, 0.29)	0.02 (0.01, 0.10)		1.00	0.34 (0.11, 1.05)	0.22 (0.09, 0.54)	0.10 (0.02, 0.41)	
Diabetes					0.138					0.391
No	1.00	0.46 (0.30, 0.71)	0.13 (0.08, 0.23)	0.12 (0.06, 0.25)		1.00	0.38 (0.22, 0.66)	0.22 (0.13, 0.38)	0.16 (0.10, 0.25)	
With	1.00	0.43 (0.20, 0.94)	0.24 (0.10, 0.56)	0.03 (0.01, 0.16)		1.00	0.75 (0.19, 3.03)	0.58 (0.14, 2.31)	0.38 (0.07, 2.14)	
Dyslipidemia					0.588					0.358
No	1.00	0.56 (0.31, 1.00)	0.15 (0.08, 0.30)	0.14 (0.06, 0.32)		1.00	0.41 (0.22, 0.76)	0.23 (0.10, 0.51)	0.22 (0.11, 0.44)	
With	1.00	0.38 (0.20, 0.70)	0.14 (0.07, 0.29)	0.09 (0.02, 0.33)		1.00	0.44 (0.15, 1.31)	0.27 (0.10, 0.75)	0.07 (0.01, 0.33)	

CI, confidence interval; OR, odds ratio.

Models were adjusted for NHANES cycles, age (continuous, year), sex (male and female), race (Hispanic, non-Hispanic white, non-Hispanic black, and others), education (below high school, high school, and college and above), marital status (married, separated, and never married), poverty-to-income ratio (<1.3, 1.3-3.5, and >3.5), body mass index (continuous, kg/m^2^), cigarette smoke (never, ever, and current), alcohol consumption (none, moderate, and heavy), physical activity (light, moderate, and heavy), hypertension (yes and no), diabetes (yes and no), and dyslipidemia (yes and no).

a *P* for interaction was estimated using the Wald test by including a multiplicative interaction term of healthy sleep score with stratified factor in the fully adjusted model.

The findings reported in the initial analysis remained strong and reliable in the subsequent sensitivity studies. When assessing the linear association between the healthy sleep score and the depressive score, we observed that for every 1-point increase in the healthy sleep score, there was a corresponding decrease of 0.99 points in the depressive score (*β*: -0.99; 95% CI: -1.16, -0.83). This association remained significant even after controlling for potential covariates (**Supplementary Table 2**). In addition, although the significance was marginal, a higher healthy sleep score remained related to a reduced likelihood of having suicidal ideation after adjusting for depression in Model 3 (0.80; 0.64, 1.00; **Supplementary Table 3**). The sensitivity analysis using multiple imputation for missing sociodemographic data yielded results consistent with the primary complete-case analysis. The association between healthy sleep score and suicidal ideation remained significant with similar effect sizes in Model 3 (0.58; 0.48, 0.71; **Supplementary Table 3**), indicating that our findings are robust to missing data assumptions.

Moreover, to address the potential influence of antidepressant treatment, we performed additional sensitivity analyses by excluding participants who reported antidepressant use and by further adjusting for antidepressant use in Model 3. These analyses yield results consistent with the primary findings, further supporting the robustness of our conclusions (**Supplementary Tables 3**; **Supplementary Tables 4**).

### Associations of individual sleep behaviors with depression and suicidal ideation

The component analyses showed that depression and suicidal ideation were negatively associated with maintaining a suitable sleep duration, no frequent insomnia, and no excessive daytime sleepiness. Of note, no snoring was associated only with suicidal ideation. In Model 3, sleep 7–8 hours/day (0.48; 0.34, 0.67), no frequent insomnia (0.24; 0.16, 0.34), and no excessive daytime sleepiness (0.18; 0.13, 0.26) were related to a lower odds ratio of depression. Meanwhile, the ORs of suicidal ideation were 0.57 (0.38, 0.85) for sleep 7–8 hours/day, 0.41 (0.27, 0.62) for no frequent insomnia, 0.67 (0.45, 0.99) for no snoring, and 0.29 (0.18, 0.44) for no excessive daytime sleepiness (**Table 4**). No significant association between snoring and depression was observed (0.89; 0.59, 1.33; **Table 4**).

**Table 4:** The relationship between individual sleep behaviors and depression and suicidal ideation.

	Individual sleep behaviors: OR (95% CI)			
	Sleep duration, 7–8 hours/day	No frequent insomnia	No snoring	No excessive daytime sleepiness
Cases/participants	3,304/5,978	4,520/5,978	2,878/5,978	5,076/5,978
Depression				
Model 1	0.45 (0.32, 0.63)	0.22 (0.16, 0.32)	0.71 (0.50, 1.00)	0.18 (0.13, 0.24)
Model 2	0.47 (0.34, 0.66)	0.23 (0.16, 0.33)	0.86 (0.60, 1.24)	0.18 (0.13, 0.25)
Model 3	0.48 (0.34, 0.67)	0.24 (0.16, 0.34)	0.89 (0.59, 1.33)	0.18 (0.13, 0.26)
Suicidal ideation				
Model 1	0.54 (0.37, 0.80)	0.39 (0.27, 0.56)	0.58 (0.39, 0.87)	0.27 (0.19, 0.40)
Model 2	0.57 (0.38, 0.84)	0.40 (0.28, 0.59)	0.66 (0.46, 0.94)	0.28 (0.18, 0.41)
Model 3	0.57 (0.38, 0.85)	0.41 (0.27, 0.62)	0.67 (0.45, 0.99)	0.29 (0.18, 0.44)

CI, confidence interval; OR, odds ratio.

Model 1: adjusted for NHANES cycles, age (continuous, year), sex (male and female), race (Hispanic, non-Hispanic white, non-Hispanic black, and others), education (below high school, high school, and college and above), marital status (married, separated, and never married), and poverty-to-income ratio (<1.3, 1.3-3.5, and >3.5);

Model 2: Adjusted for body mass index (continuous, kg/m^2^), cigarette smoke (never, ever, and current), alcohol consumption (none, moderate, and heavy), physical activity (light, moderate, and heavy), and covariates adjusted in Model 1;

Model 3: Adjusted for hypertension (yes and no), diabetes (yes and no), dyslipidemia (yes and no), and covariates adjusted in Model 2.

### Mediation role of depression in the associations of healthy sleep score and individual sleep behaviors with the likelihood of suicidal ideation

The diagnostic statistics indicated that the maximum VIF values were below 5 in both the mediation and outcome models, suggesting no serious multicollinearity (**Supplementary Table 5**). However, the residuals in both models deviated from normality (high skewness and kurtosis, supported by Q-Q plots, **Supplementary Figure 2**), and the Breusch-Pagan test indicated heteroscedasticity (*P* < 0.001; **Supplementary Table 5**). Given the large sample size, the mediation models were further estimated using robust procedures, which are less sensitive to such violations.

**Figure 1:**
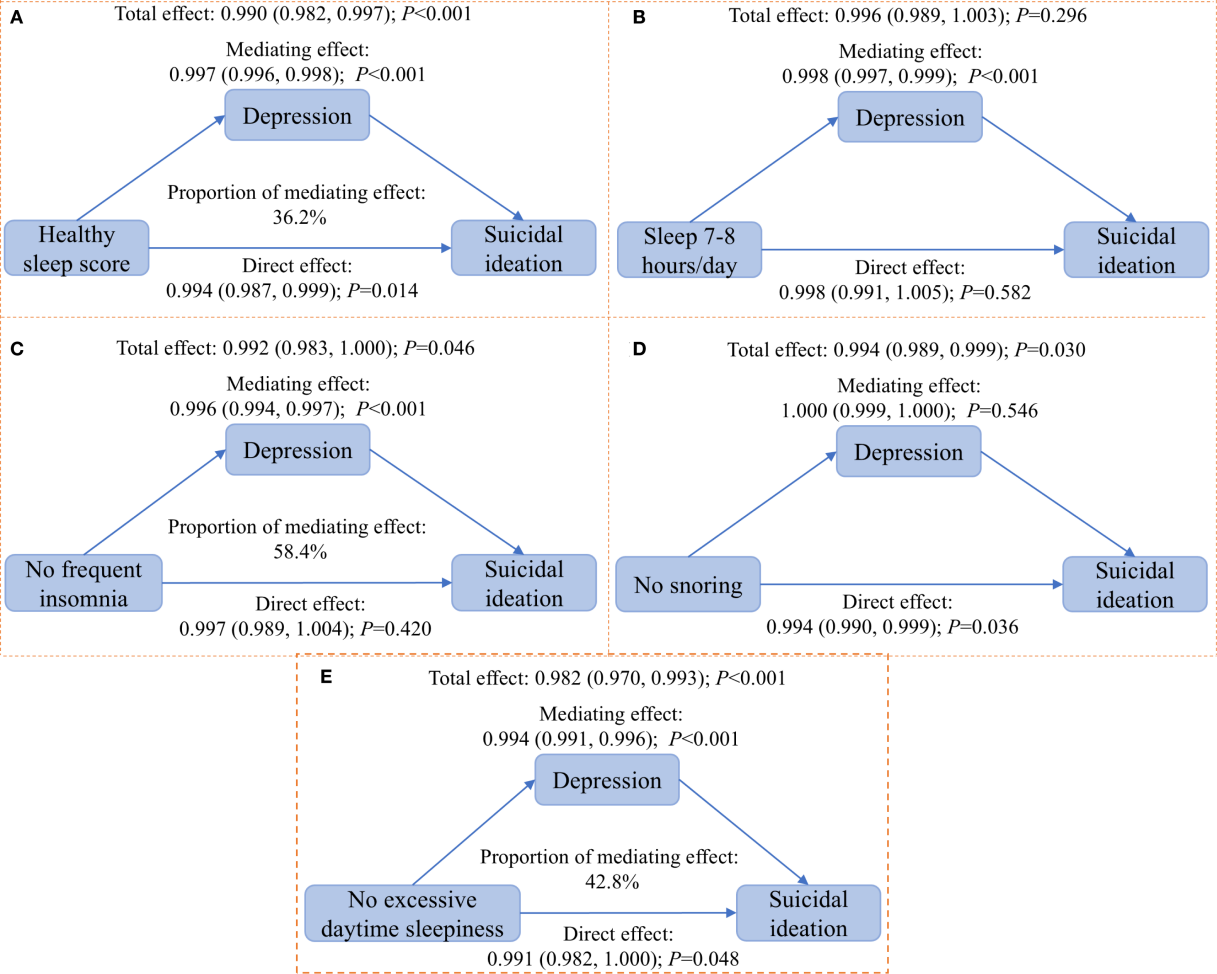
Mediation analyses of depression on the associations of the health sleep score and individual sleep behaviors with suicidal ideation. Covariates includes NHANES cycles, age (continuous, year), sex (male and female), race (Hispanic, non-Hispanic white, non-Hispanic black, and others), education (below high school, high school, and college and above), marital status (married, separated, and never married), poverty-to-income ratio (<1.3, 1.3-3.5, and >3.5), body mass index (continuous, kg/m^2^), cigarette smoke (never, ever, and current), alcohol consumption (none, moderate, and heavy), physical activity (light, moderate, and heavy), hypertension (yes and no), diabetes (yes and no), and dyslipidemia (yes and no).

The results of the mediation analyses were shown in **Figure 1**. Depression mediated the associations of the healthy sleep score, no frequent insomnia, and no excessive daytime sleepiness with suicidal ideation, with mediation proportions of 36.2% to 58.4%. No mediation role of depression was observed in the associations of sleep duration of 7–8 hours/day and no snoring with suicidal ideation. Sensitivity analysis using quasi-Bayesian approximation (5,000 simulations) confirmed the main findings (**Supplementary Table 6**).

## Discussion

This nationally representative study discovered an inverse relationship between a healthy sleep pattern—defined as 7–8 hours/day, no snoring, no frequent insomnia, and no excessive daytime sleepiness—with depression and suicidal ideation. The odds of developing depression and having suicidal ideation decreased by 52% and 42%, respectively, with every 1-point increase in the healthy sleep score. Depression partly mediated the associations of the healthy sleep score, no frequent insomnia, and no excessive daytime sleepiness with suicidal ideation.

The results of our study align with other research on the correlation between sleep patterns and depression. Both inadequate and excessive sleep length have been found to be linked to a higher likelihood of experiencing depression (26). A comprehensive meta-analysis of 34 prospective cohort studies, which included a total of 172,077 participants, revealed a noteworthy correlation between insomnia and depression (27). Additionally, significant relationships were found between depression and snoring (28) and excessive daytime sleepiness (29). To our knowledge, there are currently only two studies that have investigated the associations between depression and various sleep behaviors using the Pittsburgh Sleep Quality Index (PSQI), which evaluates sleep patterns across seven distinct dimensions. However, one of them neglected to assess the relationship between depression and the cumulative sleep score (30). Another study conducted on nursing home residents showed a positive relationship between poorer sleep quality and depression (31). Despite variations in the instruments used to evaluate sleep patterns, our research supports the consistent conclusions drawn in previous studies.

Consistent with prior investigations, our study observed a negative correlation between a healthy sleep pattern and suicidal ideation. A comprehensive cross-sectional study involving 54,948 Korean adolescents revealed a correlation between sleeping for less than 5 hours per day and an increased susceptibility to suicidal ideation (32). Self-reported insomnia (33) and daytime sleepiness (34) have both been reported to increase the likelihood of suicidal ideation in adolescents. While no specific epidemiological study has directly investigated the correlation between snoring and suicidal ideation, there is evidence indicating that symptoms such as snoring or coughing during sleep have been associated with an increased risk of suicide (35). In terms of sleep patterns, a study in the Chinese population has demonstrated that the PSQI score was positively associated with suicidal ideation (36). Similar associations were also observed in depressive outpatients in Korea (n=909) (35) and Ethiopian adults (n=1,054), even after adjusting for depression (37). Our results were robust even after accounting for antidepressant use. In addition, we determined the mediation roles of depression in the associations of sleep score, insomnia, and excessive daytime sleepiness with suicidal ideation. Although there is considerable evidence indicating that individual sleep behavior independently correlates with both depression and suicide risk, there is insufficient evidence regarding the extent to which depression mediates the relationship between sleep and suicide risk. The mediation proportions observed in our study suggested the importance of preventing suicidal ideation by decreasing depression risk among individuals with lower sleep scores. Given that depression partly mediated the association between sleep patterns and suicidal ideation, the role of adequate antidepressant treatment should also be emphasized in suicide prevention strategies.

In a general population, our study revealed a noteworthy association between following a healthy sleep pattern and depression as well as suicidal ideation and the mediation role of depression. The linear dose-response relationships found in our analyses suggested that various sleep behaviors have an additive effect on depression and suicidal ideation. Complex interactions frequently occur between these sleep behaviors (38). Changing one aspect of sleep may result in compensatory adjustments in other areas (18). For example, the duration of sleep is often shorter when insomnia occurs frequently (39), and the duration of sleep may vary depending on chronotype (40). Compared to studies that focused on individual sleep behaviors, our findings have great public health implications and are more conducive to implementing preventive interventions in the general population.

The underlying mechanisms linking sleep behaviors, depression, and suicidal ideation remain incompletely elucidated. However, Prior studies have indicated that sleep problems may contribute to depression by disrupting the regulation of mood and emotions, affecting cognitive capacities, and diminishing coping skills (41–43). In addition, sleep disturbance has been reported to be linked to the elevation of inflammatory cytokines and the dysregulation of the hypothalamic-pituitary axis, both of which are strongly linked to the development of depression (44, 45). The correlation between sleep disorders and suicidal ideation has been partially elucidated by serotonin depletion and hypothalamic-pituitary axis activation in relation to suicidal ideation (46).

The current study used an innovative indicator to evaluate the associations of comprehensive sleep behaviors with depression and suicidal ideation. There are several strengths to our study. First, our findings are highly generalizable due to the utilization of nationally representative data and a substantial sample size. Second, covariates were assessed and adjusted in the regression analyses to minimize confounders’ influence. However, certain potential restrictions should be recognized. First, the information on chronotype preference was not collected during the survey, and chronotype was not included in the assessment of sleep patterns. Considering chronotype is a significant modifiable risk factor for mental health, collecting data on chronotype in future studies is necessary. Second, the outcome assessed in our study was suicidal ideation, which is an important but not identical construct to suicidal behavior. Therefore, the findings should not be directly generalized to suicide attempts or completed suicide. Third, the study sample did not include individuals who reported taking sleeping pills, which may limit the generalizability of our results to populations with sleep medication use. Fourth, sleep and other covariates were based on self-reports, which are subject to recall bias and measurement error. Fifth, the cross-sectional design’s inherent limitations restricted the capacity for causal inference. In addition, both depressive symptoms and suicidal ideation were derived from the same instrument (PHQ-9). Although the suicidal ideation item of the PHQ-9 has been widely validated and is commonly used in large-scale epidemiological studies as a proxy for suicidal thoughts (47), relying on a single item from the same questionnaire may introduce common method bias and limit the ability to fully capture the complexity of suicidality. Therefore, our findings should be interpreted with caution, and future studies using independent and more comprehensive measures of suicidal ideation and behaviors are warranted.

## Conclusions

Our study concludes that maintaining a healthy sleep pattern is associated with a reduced likelihood of developing depression and suicidal ideation. These findings highlight the importance of promoting healthy sleep behaviors as a potential strategy for preventing prevalent mental health problems in the general population. Moreover, our results underscore the clinical relevance of early screening and intervention targeting both sleep and depressive symptoms. Future longitudinal and interventional studies are warranted to better establish causal relationships and to inform effective prevention and treatment strategies.

## Data Availability

Publicly available datasets were analyzed in this study. This data can be found here: the National Health and Nutrition Examination Survey website (https://wwwn.cdc.gov/nchs/nhanes/Default.aspx).
