# Supplementary material for: Depression mediates the association of healthy sleep patterns with suicidal ideation among U.S. adults
Source: Front Psychiatry. 2025 Sep 29;16:1644867. doi: 10.3389/fpsyt.2025.1644867 (PMC12515818; doi:10.3389/fpsyt.2025.1644867)
Supplement: Supplementary file 1 [file SupplementaryFile1.docx]

**Supplementary materials**

**Depression mediates the association of healthy sleep patterns with suicidal ideation among U.S. adults**

**Supplementary Tables**

Supplementary Table 1 Characteristics of study participants in NHANES, 2005-2008 (N=5,978)

Supplementary Table 2 The association of healthy sleep score with depressive score in NHANES, 2005-2008

Supplementary Table 3 The association between healthy sleep score and suicidal ideation independent of depression/antidepresant use and in imputed dataset in NHANES, 2005-2008

Supplementary Table 4 The association of healthy sleep score with depression and suicidal ideation among individuals without antidepressant use (N = 5,600)

Supplementary Table 5 Diagnostic statistics for assumption checks in the mediation and outcome models

Supplementary Table 6 Mediation analysis of sleep pattern on suicidal ideation via depression using quasi-Bayesian approximation (β and 95% confidence interval)

**Supplementary Figures**

Supplementary Figure 1 Flowchart of the participants selection

Supplementary Figure 2 Q-Q plots of residuals. (A) for mediation models; (B) for outcome models

**Supplementary Tables**

**Supplementary Table 1** Characteristics of study participants in NHANES, 2005-2008 (N=5,978)

| Characteristics | Overall | Healthy sleep score | | | | *P* value ^a^ |
| --- | --- | --- | --- | --- | --- | --- |
|  |  | 0-1 | 2 | 3 | 4 |  |
| No. of participants, n (%) | 5,978 (100.00) | 850 (14.22) | 1,568 (26.23) | 2,251 (37.65) | 1,309 (21.90) |  |
| Age, years | 45.00 ± 0.44 | 45.09 ± 0.52 | 45.42 ± 0.63 | 45.44 ± 0.48 | 43.54 ± 0.67 | 0.076 |
| Sex, n (%) |  |  |  |  |  | 0.002 |
| Male | 3,129 (52.82) | 436 (51.31) | 852 (56.60) | 1,214 (53.78) | 627 (47.74) |  |
| Female | 2,849 (47.18) | 414 (48.69) | 716 (43.40) | 1,037 (46.22) | 682 (52.26) |  |
| Race, n (%) |  |  |  |  |  | 0.006 |
| Hispanic | 1,586 (12.69) | 199 (10.27) | 389 (11.73) | 612 (13.21) | 386 (14.40) |  |
| Non-Hispanic white | 2,885 (71.10) | 424 (72.93) | 719 (69.20) | 1,083 (71.08) | 659 (72.22) |  |
| Non-Hispanic black | 1,278 (10.66) | 195 (12.15) | 397 (12.95) | 472 (10.25) | 214 (7.80) |  |
| Others | 229 (5.55) | 32 (4.66) | 63 (6.13) | 84 (5.46) | 50 (5.58) |  |
| Education, n (%) |  |  |  |  |  | <0.001 |
| Below high school | 1,671 (18.14) | 252 (20.83) | 442 (18.25) | 624 (18.10) | 353 (16.41) |  |
| High school | 1,428 (24.08) | 242 (31.47) | 388 (25.08) | 514 (22.87) | 284 (20.44) |  |
| College and above | 2,879 (57.78) | 356 (47.7) | 738 (56.67) | 1,113 (59.03) | 672 (63.14) |  |
| Marital status, n (%) |  |  |  |  |  | <0.001 |
| Married | 3,461 (60.72) | 450 (54.89) | 904 (59.58) | 1,379 (64.23) | 728 (59.68) |  |
| Separated | 1,595 (23.54) | 276 (30.78) | 431 (24.61) | 546 (20.96) | 342 (22.20) |  |
| Never married | 922 (15.74) | 124 (14.33) | 233 (15.81) | 326 (14.81) | 239 (18.12) |  |
| PIR, n (%) |  |  |  |  |  | 0.003 |
| <1.3 | 1,610 (17.32) | 277 (22.83) | 407 (16.75) | 567 (15.97) | 359 (16.88) |  |
| 1.3-3.5 | 2,387 (37.07) | 337 (39.01) | 652 (39.06) | 906 (36.32) | 492 (34.85) |  |
| >3.5 | 1981 (45.61) | 236 (38.16) | 509 (44.19) | 778 (47.70) | 458 (48.27) |  |
| Body mass index, kg/m^2^ | 28.68 ± 0.18 | 31.00 ± 0.35 | 29.35 ± 0.20 | 28.57 ± 0.20 | 26.68 ± 0.22 | <0.001 |
| Obesity status, n (%) |  |  |  |  |  | <0.001 |
| Normal | 1,705 (31.10) | 168 (19.50) | 386 (26.80) | 635 (30.27) | 516 (44.56) |  |
| Overweight | 2,122 (34.89) | 276 (34.49) | 524 (34.32) | 841 (35.78) | 481 (34.29) |  |
| Obesity | 2,151 (34.01) | 406 (46.02) | 658 (38.88) | 775 (33.95) | 312 (21.15) |  |
| Cigarette smoke, n (%) |  |  |  |  |  | <0.001 |
| Never | 3,215 (53.31) | 370 (42.28) | 815 (51.19) | 1,215 (53.38) | 815 (62.42) |  |
| Ever | 1,488 (23.91) | 224 (24.77) | 383 (22.81) | 575 (24.93) | 306 (22.91) |  |
| Current | 1,275 (22.78) | 256 (32.94) | 370 (26.00) | 461 (21.69) | 188 (14.68) |  |
| Alcohol consumption, n (%) | |  |  |  |  | 0.080 |
| None | 1,760 (24.81) | 234 (24.23) | 460 (23.68) | 644 (24.10) | 422 (27.68) |  |
| Moderate | 3,706 (65.32) | 542 (65.88) | 962 (65.08) | 1,403 (65.55) | 799 (64.87) |  |
| Heavy | 512 (9.86) | 74 (9.88) | 146 (11.24) | 204 (10.34) | 88 (7.45) |  |
| Physical activity, n (%) |  |  |  |  |  | 0.060 |
| Light | 2,307 (32.97) | 353 (37.55) | 582 (32.02) | 881 (33.58) | 491 (30.22) |  |
| Moderate | 1,763 (32.88) | 217 (28.12) | 469 (33.35) | 684 (33.79) | 393 (33.72) |  |
| Heavy | 1,908 (34.15) | 280 (34.33) | 517 (34.63) | 686 (32.63) | 425 (36.06) |  |
| Hypertension, yes, n (%) | 2,533 (37.41) | 412 (44.20) | 718 (39.43) | 958 (37.97) | 445 (29.97) | <0.001 |
| Diabetes, yes, n (%) | 877 (10.47) | 185 (15.78) | 238 (10.99) | 322 (9.97) | 132 (7.48) | <0.001 |
| Dyslipidemia, yes, n (%) | 2,649 (42.38) | 414 (48.87) | 713 (42.97) | 1,056 (45.26) | 466 (32.84) | <0.001 |

Abbreviation: NHANES, National Health and Nutrition Examination Survey; PIR, poverty-to-income ratio.

^a^ *P* values were calculated using Rao–Scott Chi-square test and analysis of variance for categorical and continuous variables, respectively.

Data was presented as mean ± standard error (continuous variables) and number (percent) (categorical variables).

**Supplementary Table 2** The association of healthy sleep score with depressive score in NHANES, 2005-2008

|  | Healthy sleep score: *β* (95% CI) | | | | *P* for trend | Per 1 point increment |
| --- | --- | --- | --- | --- | --- | --- |
|  | 0-1 | 2 | 3 | 4 |  |  |
| Depressive score |  |  |  |  |  |  |
| Participants | 850 | 1,568 | 2,251 | 1,309 | - | - |
| Model 1 | 0 (reference) | -2.06 (-2.55, -1.56) | -2.96 (-3.49, -2.42) | -3.41 (-3.90, -2.92) | <0.001 | -1.04 (-1.19, -0.89) |
| Model 2 | 0 (reference) | -1.99 (-2.51, -1.47) | -2.87 (-3.43, -2.32) | -3.24 (-3.75, -2.72) | <0.001 | -1.00 (-1.15, -0.84) |
| Model 3 | 0 (reference) | -1.98 (-2.54, -1.42) | -2.86 (-3.45, -2.26) | -3.22 (-3.78, -2.66) | <0.001 | -0.99 (-1.16, -0.83) |

Abbreviation: CI, confidence interval; NHANES, National Health and Nutrition Examination Survey.

Model 1: adjusted for NHANES cycles, age (continuous, year), sex (male and female), race (Hispanic, non-Hispanic white, non-Hispanic black, and others), education (below high school, high school, and college and above), marital status (married, separated, and never married), and poverty-to-income ratio (<1.3, 1.3-3.5, and >3.5);

Model 2: Adjusted for body mass index (continuous, kg/m^2^), cigarette smoke (never, ever, and current), alcohol consumption (none, moderate, and heavy), physical activity (light, moderate, and heavy), and covariates adjusted in Model 1;

Model 3: Adjusted for hypertension (yes and no), diabetes (yes and no), dyslipidemia (yes and no), and covariates adjusted in Model 2.

**Supplementary Table 3** The association between healthy sleep score and suicidal ideation independent of depression/antidepresant use and in imputed dataset in NHANES, 2005-2008

|  | Healthy sleep score: *β* (95% CI) | | | | *P* for trend | Per 1 point increment |
| --- | --- | --- | --- | --- | --- | --- |
|  | 0-1 | 2 | 3 | 4 |  |  |
| Cases/participants | 56/850 | 48/1,568 | 42/2,251 | 16/1,309 | - | - |
| Model 1 | 1.00 (reference) | 0.43 (0.24, 0.77) | 0.26 (0.15, 0.44) | 0.18 (0.10, 0.31) | <0.001 | 0.56 (0.47, 0.68) |
| Model 2 | 1.00 (reference) | 0.43 (0.24, 0.79) | 0.27 (0.16, 0.46) | 0.20 (0.12, 0.33) | <0.001 | 0.57 (0.48, 0.68) |
| Model 3 | 1.00 (reference) | 0.45 (0.23, 0.88) | 0.28 (0.15, 0.50) | 0.20 (0.12, 0.36) | <0.001 | 0.58 (0.48, 0.70) |
| Model 3 + depression | 1.00 (reference) | 0.67 (0.25, 1.79) | 0.63 (0.31, 1.30) | 0.49 (0.23, 1.03) | 0.039 | 0.80 (0.64, 1.00) |
| Model 3 + antidepressant | 1.00 (reference) | 0.45 (0.23, 0.88) | 0.28 (0.15, 0.50) | 0.20 (0.12, 0.36) | <0.001 | 0.58 (0.48, 0.70) |
| Model 3 with Imputed dataset | 1.00 (reference) | 0.45 (0.22, 0.92) | 0.28 (0.15, 0.53) | 0.20 (0.11, 0.37) | <0.001 | 0.58 (0.48, 0.71) |

Abbreviation: CI, confidence interval; NHANES, National Health and Nutrition Examination Survey; OR, odds ratio.

Model 1: adjusted for NHANES cycles, age (continuous, year), sex (male and female), race (Hispanic, non-Hispanic white, non-Hispanic black, and others), education (below high school, high school, and college and above), marital status (married, separated, and never married), and poverty-to-income ratio (<1.3, 1.3-3.5, and >3.5);

Model 2: Adjusted for body mass index (continuous, kg/m^2^), cigarette smoke (never, ever, and current), alcohol consumption (none, moderate, and heavy), physical activity (light, moderate, and heavy), and covariates adjusted in Model 1;

Model 3: Adjusted for hypertension (yes and no), diabetes (yes and no), dyslipidemia (yes and no), and covariates adjusted in Model 2.

**Supplementary Table 4** The association of healthy sleep score with depression and suicidal ideation among individuals without antidepressant use (N = 5,600)

|  | Healthy sleep score: OR (95% CI) | | | | *P* for trend | Per 1 point increment |
| --- | --- | --- | --- | --- | --- | --- |
|  | 0-1 | 2 | 3 | 4 |  |  |
| Depression |  |  |  |  |  |  |
| Cases/participants | 113/762 | 88/1,456 | 59/2,139 | 22/1,243 | - | - |
| Model 1 | 1.00 | 0.40 (0.29, 0.57) | 0.15 (0.08, 0.25) | 0.11 (0.06, 0.22) | <0.001 | 0.47 (0.40, 0.55) |
| Model 2 | 1.00 | 0.41 (0.28, 0.60) | 0.14 (0.08, 0.26) | 0.12 (0.06, 0.25) | <0.001 | 0.47 (0.40, 0.56) |
| Model 3 | 1.00 | 0.41 (0.27, 0.62) | 0.15 (0.08, 0.28) | 0.12 (0.06, 0.27) | <0.001 | 0.48 (0.40, 0.57) |
| Suicidal ideation |  |  |  |  |  |  |
| Cases/participants | 41/762 | 42/1,456 | 39/2,139 | 14/1,243 | - | - |
| Model 1 | 1.00 | 0.48 (0.29, 0.82) | 0.30 (0.17, 0.52) | 0.21 (0.11, 0.38) | <0.001 | 0.60 (0.50, 0.72) |
| Model 2 | 1.00 | 0.49 (0.28, 0.84) | 0.31 (0.17, 0.54) | 0.23 (0.14, 0.39) | <0.001 | 0.62 (0.53, 0.73) |
| Model 3 | 1.00 | 0.50 (0.28, 0.92) | 0.32 (0.17, 0.60) | 0.24 (0.14, 0.41) | <0.001 | 0.63 (0.53, 0.74) |

Abbreviation: CI, confidence interval; NHANES, National Health and Nutrition Examination Survey; OR, odds ratio.

Model 1: adjusted for NHANES cycles, age (continuous, year), sex (male and female), race (Hispanic, non-Hispanic white, non-Hispanic black, and others), education (below high school, high school, and college and above), marital status (married, separated, and never married), and poverty-to-income ratio (<1.3, 1.3-3.5, and >3.5);

Model 2: Adjusted for body mass index (continuous, kg/m^2^), cigarette smoke (never, ever, and current), alcohol consumption (none, moderate, and heavy), physical activity (light, moderate, and heavy), and covariates adjusted in Model 1;

Model 3: Adjusted for hypertension (yes and no), diabetes (yes and no), dyslipidemia (yes and no), and covariates adjusted in Model 2.

**Supplementary Table 5** Diagnostic statistics for assumption checks in the mediation and outcome models

| Model | VIF (max) | Skewness (residuals) | Kurtosis (residuals) | BP test *P* |
| --- | --- | --- | --- | --- |
| Mediation model  (depression~sleep pattern) | 2.90 | 4.21 | 23.09 | <0.001 |
| Outcome model  (Suicidal ideation~sleep pattern+depression) | 3.49 | 6.43 | 66.39 | <0.001 |

Abbreviation: BP, Breusch-Pagan; VIF, variance inflation factor.

**Supplementary Table 6** Mediation analysis of sleep pattern on suicidal ideation via depression using quasi-Bayesian approximation (*β* and 95% confidence interval)

| Exposures | Mediator | Outcomes | Total effect | Mediating effect | Direct effect | Proportion of mediating effect |
| --- | --- | --- | --- | --- | --- | --- |
| Healthy sleep score |  |  | -0.010*** | -0.003*** | -0.007* | 35.7%*** |
| Sleep 7-8 hours/day |  |  | -0.004 | -0.002*** | -0.002 | 34.2% |
| No frequent insomnia | Depression | Suicidal ideation | -0.008* | -0.005*** | -0.004 | 56.1%* |
| No snoring |  |  | -0.006* | -0.0002 | -0.006* | 3.7% |
| No excessive daytime sleepiness |  |  | -0.018*** | -0.008*** | -0.011* | 42.1%*** |

***, *P* <0.001; **, 0.001≤ *P* <0.01; *, 0.01≤ *P* <0.05.

Models were adjusted for NHANES cycles, age (continuous, year), sex (male and female), race (Hispanic, non-Hispanic white, non-Hispanic black, and others), education (below high school, high school, and college and above), marital status (married, separated, and never married), poverty-to-income ratio (<1.3, 1.3-3.5, and >3.5), body mass index (continuous, kg/m^2^), cigarette smoke (never, ever, and current), alcohol consumption (none, moderate, and heavy), physical activity (light, moderate, and heavy), hypertension (yes and no), diabetes (yes and no), and dyslipidemia (yes and no).

.
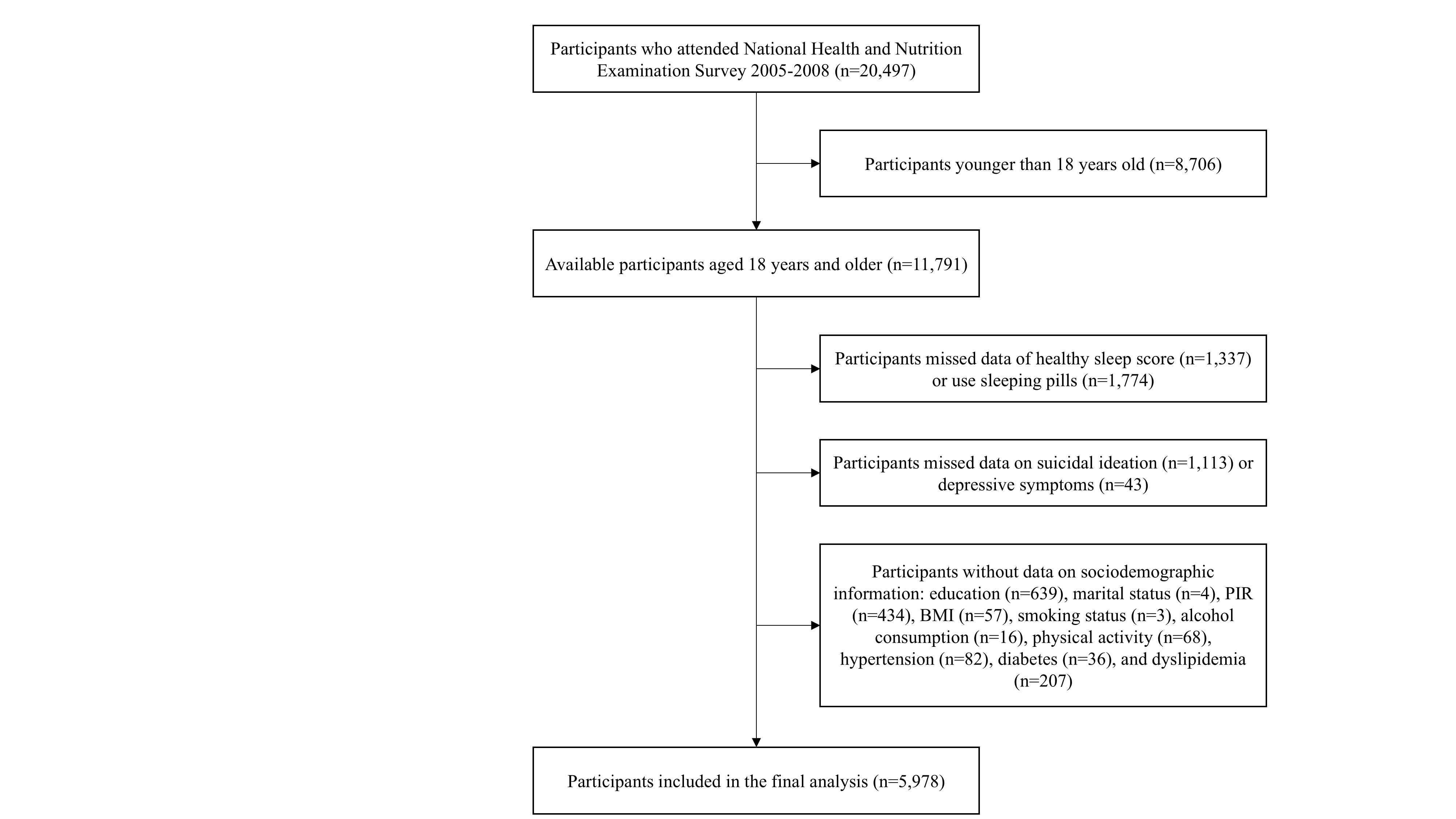


**Supplementary Figure 1** Flowchart of the participants selection

**
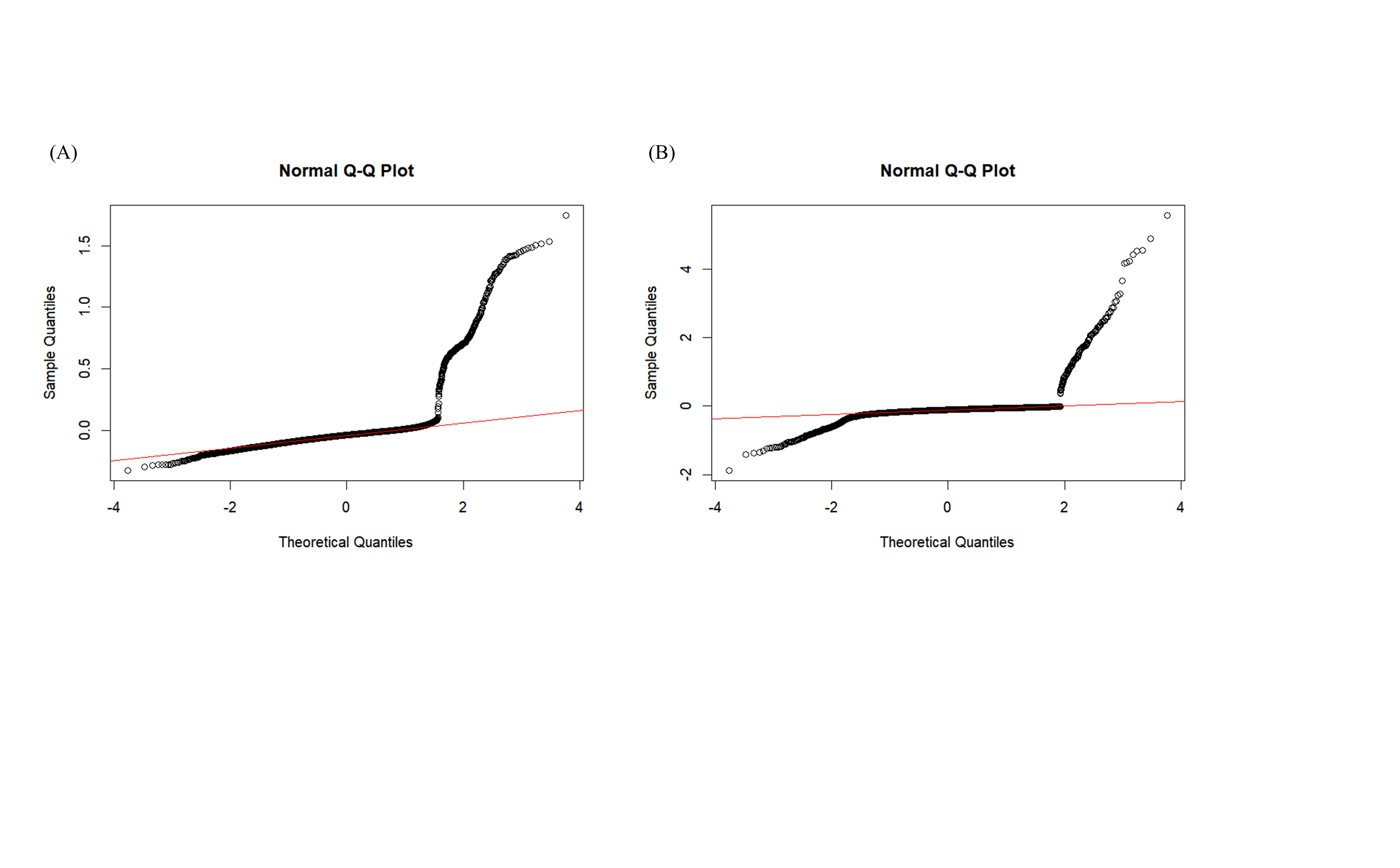
Supplementary Figure 2** Q-Q plots of residuals. (A) for mediation models; (B) for outcome models
